# Analysis of the Relationship between Psychiatric and Addiction-Related Disorders in Patients of an Outpatient Addiction Treatment Clinic for Children and Adolescents

**DOI:** 10.3390/children11040414

**Published:** 2024-03-31

**Authors:** Tomas Jandac, Lenka Stastna

**Affiliations:** 1 Department of Addictology, First Faculty of Medicine, Charles University, 128 00 Prague, Czech Republic; lenka.stastna@lf1.cuni.cz; 2 Department of Addictology, General University Hospital in Prague, 128 00 Prague, Czech Republic

**Keywords:** paedopsychiatry, addiction medicine, dual diagnosis, paediatric medicine

## Abstract

Introduction: Dual diagnosis is used in addiction medicine to refer to the co-occurrence of an addiction-related disorder and another psychiatric disorder in the same individual. Adolescence is a key period for the development of both mental disorders and addictions. Objective: The aim of this study is to describe the relationships between psychiatric and addiction-related disorders in patients of the Outpatient Addiction Treatment Clinic for Children and Adolescents at the 1st Faculty of Medicine, Charles University in Prague in 2015–2022. Methods: Data were retrospectively analyzed from the hospital’s medical system, which collects basic diagnostic data on patients. Descriptive statistics and cluster analysis were performed to identify relationships between psychiatric and addiction-related disorders. Results: Of the 450 patients, 153 patients (34%) met the criteria for dual diagnosis. The most common addiction-related disorders were mental and behavioural disorders due to the use of cannabinoids (35%) and internet gaming disorder (35%). The most common psychiatric diagnoses were behavioural and emotional disorders with usual onset in childhood and adolescence (64%), with a lower prevalence in girls than in boys. Conclusions: These findings may be important for the diagnosis and treatment of risky behaviours and addictions in children and adolescents.

## 1. Introduction

Dual diagnosis is a common term in addiction medicine, but it is often used inconsistently. The World Health Organization defines dual diagnosis as the co-occurrence in the same individual of a psychoactive substance use disorder and another psychiatric disorder [1]. Clinical case observations raise the concern of whether mental disorder can increase the risk of addiction, or whether addiction increases the risk of mental disorder, or whether mental illness and addiction are expressions of a single underlying cause [2].

Adolescence is an important developmental period which is typically associated with behavioural aspects such as excitability, impulsivity, reward preference, and a tendency to spend leisure time with peers. While such a context may play a positive role in personal development, it can also be a contributory factor for the development of risk behaviours, including substance use. However, it is important to understand the patterns of substance use behaviour, which may range from occasional experimentation to habituation and dependence [3]. The period of adolescence is key for the development of the areas of the brain responsible for the maturing of personality and independent skills, but it is also a period in which symptoms of mental disorders may begin to manifest themselves. Mental disorders associated with dual diagnoses include anxiety disorders, depressive disorders, psychotic disorders, bipolar affective disorder, and antisocial personality disorder. While these associations have been explored in relation to adult individuals, many of the mental health disorders under consideration begin to develop during adolescence [4]. A four-year follow-up study of 627 secondary school students upheld the hypothesis that mental disorders predict the development of substance use disorders. Post-traumatic stress disorder predicted substance use in general, while social phobia predicted alcohol use disorder. None of the anxiety disorders predicted non-alcohol substance use disorders. Depressive disorders predicted alcohol use disorders. Substance use disorders did not predict any subsequent development of either anxiety or depressive disorders. Alcohol use disorders, on the other hand, were associated with a higher probability of obsessive-compulsive disorder [5]. To a greater degree than the adult population, adolescents tend to engage in polydrug use patterns. Nevertheless, the majority of the studies concerned with prevention and treatment interventions focus on individual substances. In addition, some studies [6,7,8] demonstrate that polysubstance use is more common among individuals with mental health comorbidities. Studies also suggest that mental health conditions such as conduct disorders and attention deficit hyperactivity disorders correlate with multiple substance use. The aetiology and prognosis of addictive disorders among adolescents can hardly be understood without the correct identification of their patterns of use and the related risks. A systematic review of studies exploring dual diagnoses among children and adolescents who were primarily treated for mental illness found that the most frequent psychiatric comorbidities were affective disorders, conduct disorders, anxiety disorders, and psychotic disorders [9]. The treatment of adolescents with co-occurring psychiatric disorders may show lower effectiveness. Early and thorough assessment and referral for appropriate treatment are therefore essential if treatment outcomes are to be improved [10,11,12,13,14]. A systematic review was used to determine the factors responsible for the decline in the prevalence of the use of certain substances in the target population of children and adolescents over the past 25 years. These factors included a lower degree of adverse childhood experiences, changes in parenting styles, and the early treatment of mental health disorders among children and adolescents [15]. The outpatient addiction treatment service for children and adolescents is the fifth specialized service of The Department of Addictology at Charles University’s First Faculty of Medicine. The programme was created after evaluating the needs of Prague and Central Bohemia. Patients and professionals in related fields welcomed the addition to the existing network of services. This outpatient service builds on the legacy of a specialized facility for youth founded by Professor Mečíř in 1957. Pilot testing confirmed the target age group, which includes children from 12 years old upwards and adolescents up to 18. It primarily focuses on substance use disorders (F.10–F.19 in the ICD-10 code) and conditions related to behavioural addictions [16]. The codes F10–F19 related to mental and behavioural disorders due to psychoactive substance use in ICD-10 [17].

Despite being a significant problem for public health and causing strain on social services, the co-occurrence of mental health conditions and substance abuse in young people has not received as much research attention as it deserves. This situation creates a pressing need for policymakers and mental health professionals to address this issue. Research has clearly shown that the use of psychoactive substances can be linked to the presence or development of various mental health problems in adults. These conditions often occur together, making it difficult to determine which came first. While this comorbidity is well-documented among adults, the situation for children and adolescents remains a significant knowledge gap. There is a lack of in-depth research on how psychoactive substances might influence the development or presence of mental health problems in younger age groups [18]. In particular, the aim of this study is to determine whether there is a relationship between the occurrence of a particular type of addiction-related disorder and the occurrence of a type of dual diagnosis in the target group.

## 2. Materials and Methods

### 2.1. Data Collection

The data were extracted from the FONS hospital system utilised by the Outpatient Addiction Treatment Clinic for Children and Adolescents (ADDA). It did not contain any variables which would make it possible to identify the patients. The data had been fed into the system since 2015. Data up to 2022 were utilised in the present study. The system can be accessed during each patient’s visit to the clinic, with the relevant data being entered by authorised staff. Consent for the study was given by all patients (their parents, respectively) at the beginning of the care in the outpatient service. This was part of the ethical committee approval for the study.

The FONS database record contains the following variables: gender, year of birth, principal diagnosis, and other psychiatric diagnoses (1, 2, 3, 4). These data were used to create a data matrix, by means of which visits to the ADDA had to be matched with individual patients to prevent multiplication of the sample. Throughout the data collection process, all the patients were diagnosed by the same physician. The diagnostic process in its entirety was supported by the supervision of another physician and by diagnostic background work by two psychologists. Diagnoses were determined according to the International Classification of Diseases, 10th Revision [17].

### 2.2. Study Sample

The sample comprises all the patients who have sought the services of the Outpatient Addiction Treatment Clinic for Children and Adolescents (ADDA) based at the Department of Addictology of the 1st Faculty of Medicine of Charles University and the General University Hospital in Prague. The sample was recruited from the study population by means of institutional sampling. The total number of ADDA patients from 2015 to 2022 was 450. Girls accounted for 33.8% of the sample and boys for 66.2%. Out of the total of 450 patients, 57 were diagnosed with no addiction-related disorder. There were thus altogether 393 patients with addiction-related disorders. The prevalence of dual diagnoses in this sample of patients was 38.9%, with 31.8% of the patients having one co-occurring psychiatric diagnosis and 7% being diagnosed with two co-occurring mental health conditions. The final sample therefore comprised 153 patients of the clinic with psychiatric comorbidities.

### 2.3. Data Analysis

The data were used for descriptive statistics which made it possible to present the prevalence of dual diagnoses in the study population and other diagnostic information. On the basis of data characteristics, the patients’ diagnostic data were allotted to sets, with the structure of such sets not being pre-determined. In this sense, the structure emerged as a system of categories ranging from selecting subjects on the basis of similarity to various categories associating dissimilar subjects. The objective is to look for natural groups of subjects. Using cluster analysis, we proposed a categorising structure reflecting the proportions of the data. Cluster analysis is an exploratory multidimensional statistical method which we used to generate clusters composed of mutually similar categories of patients with dual diagnoses. For each group, the youngest and oldest age of the patients in the group and the most frequently represented addiction-related disorder and mental health comorbidity were determined. In addition, each group was defined by a silhouette coefficient, which ranges from −1 to +1. The silhouette coefficient is a metric that measures how well each data point fits into its assigned cluster. It combines information about both the cohesion (how close a data point is to other points in its own cluster) and the separation (how far a data point is from points in other clusters) of the data point. The higher its value is and the nearer it draws to +1, the closer the subjects within the group are to each other and the more different they are from those outside the group [19]. Model-based clustering with multiple variables was used. The analysis was performed in the software RStudio.

## 3. Results

### 3.1. Distribution of Psychiatric Comorbidities

The most common addiction-related disorders were mental and behavioural disorders due to the use of cannabinoids (F12.2) and other habit and impulse disorders (F63.8), with each being found among 34.9% of the patients. The third most common addiction-related disorder was polydrug use (F19.2), found among 18.8% of the patients, followed by stimulant use disorders (F15.2) (14.5%) and alcohol use disorders (F10.2) (13.2%). Disorders resulting from the use of tobacco were diagnosed in 5.3% of the patients and those caused by the use of sedatives and hypnotics in 1% of the patients. The most common comorbid mental health conditions were those categorised under behavioural and emotional disorders with onset usually occurring in childhood and adolescence (F90–F98), which were identified in 64.1% of the individuals. Disturbance of activity and attention (F90.0) accounted for 22%, hyperkinetic conduct disorder (F90.1) for 31%, and unsocialised conduct disorder (F91.1) for 29.4% of the cases, with other conduct disorders accounting for 17.6% of the cases within the conduct disorders segment. The block of disorders which is the second most commonly represented in the sample covers disorders of adult personality and behaviour (F60–F69), excluding pathological gambling and other habit and impulse disorders. This segment of disorders was identified among 15.5% of the individuals. The third most common group involves neurotic, stress-related, and somatoform disorders (F40–F49), which were found in 11.3% of the individuals. Behavioural syndromes associated with physiological disturbances and physical factors (F50–F59), disorders of psychological development (F80–F89), and mood (affective) disorders (F30–F39) were also identified (Table 1). This proportionate representation of disorders is more accurate in reflecting the rates among the boys. The girls, in comparison, showed a lower prevalence (34.9%) of behavioural and emotional disorders with onset usually occurring in childhood and adolescence (F90–F98), but higher rates of disorders of adult personality and behaviour (F60–F69) (22.2%), behavioural syndromes associated with physiological disturbances and physical factors (F50–F59) (15.9%), neurotic, stress-related, and somatoform disorders (F40–F49) (14.3%), and mood (affective) disorders (F30–F39) (7.9%).

### 3.2. The Relationship between Types of Addiction-Related Disorders and Dual Diagnoses

Detailed summaries of the representation of psychiatric diagnoses among patients with dual diagnoses are provided in Table 2 and Table 3, with the latter also providing a gender-specific breakdown. The above data indicate that the greatest share of psychiatric diagnoses comes under behavioural and emotional disorders with onset usually occurring in childhood and adolescence. We divided this block into three segments: hyperkinetic disorders (F900–F909), conduct disorders, including mixed disorders of conduct and emotions, which are not diagnosed according to ICD-10 unless conduct disorders are present (a combination with a conduct disorder is required)—the second group thus covers diagnoses F910–F929—and the remaining segment of behavioural and emotional disorders with onset usually occurring in childhood and adolescence. This latter segment involves the F93–F98 area, which includes emotional disorders with onset specific to childhood, disorders of social functioning with onset specific to childhood and adolescence, tic disorders, and other behavioural and emotional disorders.

Mental and behavioural disorders due to the use of alcohol were those that co-occurred most frequently with psychiatric diagnoses belonging to the disorders of adult personality and behaviour block (one boy and three girls). Conduct disorders and mood disorders were each found in two individuals. Mood disorders were diagnosed only in girls, while conduct disorders were found in one boy and one girl.

Mental and behavioural disorders due to the use of cannabinoids co-occurred most frequently with psychiatric diagnoses from the conduct disorders group (13 individuals), which were more likely to appear among the boys than among the girls (nine vs. four). The second most common group of psychiatric conditions was hyperkinetic disorders (10 individuals), which were present only among boys. Disorders of adult personality and behaviour (five individuals), which were more likely to occur among girls (three girls vs. two boys) were the third most common area of mental health conditions. Five individuals (boys only) were also diagnosed with other habit and impulse disorders.

Mental and behavioural disorders due to the use of stimulants were most likely to co-occur with psychiatric diagnoses from among the conduct disorders group (one boy and two girls); two girls were diagnosed with a neurotic, stress-related, and somatoform disorder, two girls with disorders grouped under behavioural syndromes associated with physiological disturbances, two boys had hyperkinetic disorders, and two girls were diagnosed with behavioural and emotional disorders with onset usually occurring in childhood and adolescence.

The psychiatric comorbidities that most commonly co-occurred with mental and behavioural disorders resulting from the use of multiple psychoactive substances were conduct disorders (seven girls and six boys), hyperkinetic disorders (12 individuals, boys only), and neurotic, stress-related, and somatoform disorders (four girls and three boys).

Our research question was also addressed by means of a multidimensional exploratory statistical method, specifically cluster analysis. Eight groups of mutually similar individuals (clusters) were generated using this method, with each group containing the most frequently represented addiction-related disorder and psychiatric disorder. 

In the first group, the youngest age was 13 and the oldest 17, with the average age being 16.1 and 17 being the most common age. The silhouette value was 0.25. Addiction-related diagnoses were represented by mental and behavioural disorders resulting from multiple drug use (F19, five individuals) and psychiatric conditions were from among behavioural syndromes associated with physiological disturbances and physical factors (F50–F59). 

In the second group, the youngest and oldest ages were 13 and 18 years, the average age was 16.1, and the most common age was 16. The silhouette value was determined at 0.24. The addiction-related diagnosis was mental and behavioural disorders resulting from multiple drug use (F19, 14 individuals) and the psychiatric condition was a disorder from the conduct disorders category (F91). 

In the third group, the youngest age was nine and the oldest 18 years, with the average being 16.9 and the most frequent age 17. The silhouette value was determined at 0.24. The addiction-related diagnosis was mental and behavioural disorders due to the use of cannabinoids (F12, 20 individuals), with the psychiatric comorbidity being a disorder from the conduct disorders group (F91). 

In the fourth group, the youngest age was 14 and the oldest 18, with the average being 16.7 years and 17 being the most frequent age. The silhouette value was 0.21. The psychiatric disorder was a diagnosis belonging to the disorders of adult personality and behaviour segment (F60–F69) and the substance use disorders were divided into mental and behavioural disorders related to the use of alcohol (F10, four individuals), cannabinoids (F12, three individuals), stimulants (F15, two individuals), and multiple psychoactive substances (F19, three individuals). 

In the fifth group, the youngest age was 13 and the oldest 18, with the average being 16.7 years and the most frequent age 17. The silhouette value for this group was 0.24. The substance use diagnosis was mental and behavioural disorders resulting from multiple drug use (F19, six individuals) and psychiatric conditions were represented by conditions from the neurotic, stress-related, and somatoform disorders diagnostic range (F40–F48). 

In the sixth group, the youngest age was 8 and the oldest 18, with the average being 14.9 years and the most frequent age 16. The silhouette value was 0.24. The psychiatric disorders were from among hyperkinetic disorders (F90) and were evenly distributed across addiction-related disorders, specifically mental and behavioural disorders due to the use of alcohol (F10) and stimulants (F15) and other habit and impulse disorders (F638). 

In the seventh group, the youngest age was 12 and the oldest 18, with the average being 16.6 years and the most frequent age 17. The silhouette value was 0.26. The addiction-related diagnosis was represented by mental and behavioural disorders resulting from polydrug use (F19, 12 individuals), while the psychiatric condition was a hyperkinetic disorder (F90). 

In the eighth group, the youngest age was 12 and the oldest 18, with the average being 15.5 years and the most frequent age 16. The silhouette value was 0.26. The addiction-related diagnosis was represented by mental and behavioural disorders resulting from the use of cannabinoids (F12, 10 individuals) and psychiatric conditions were represented by hyperkinetic disorders (F90).

## 4. Discussion

While receiving reasonable research attention as regards the adult population, the issue of dual diagnoses among children and adolescents is covered by the international literature to a much lesser degree. Moreover, the international conceptualisation of addiction studies with a focus on children and adolescents is currently on the rise. However, such activities require the relevant target population to be described and its specific characteristics and patterns of use identified. While international studies have reported significant representation of people with problems due to alcohol use, alcohol use disorder was only the fifth most common comorbidity in our target population. This difference may be due to different levels of availability of addictive substances across countries and varying habitual patterns of use of different types of substances among children and adolescents. The severity of an addiction-related disorder or the type of the substance used may also be influenced by the type of drug service that was attended, as more serious addictive disorders tend to be dealt with by other forms of services, such as residential facilities. The most common mental health comorbidities were behavioural and emotional disorders with onset usually occurring in childhood and adolescence, which were found in 64% of the participants. This finding corresponds with the conclusions drawn [6,7,8]. On the other hand, our results do not support the findings of Tejeda-Romero et al. [4]. This incongruity may be attributed to the Mexican study not being focused on young people with hyperkinetic disorders and disturbances of activity. The difference may also be due to the fact that the above studies did not take account of disorders of adult personality and behaviour, which were diagnosed in our sample as the second most frequent comorbidities. By their nature, such disorders should not be diagnosed among the population under consideration, and the practitioner may have chosen this diagnosis in consideration of the development in individuals displaying a borderline trajectory.

Further assessment of the relationship between addiction-related disorders and comorbid mental health conditions was performed using the exploratory statistical method of cluster analysis, by means of which we generated eight groups of mutually similar subjects characterised by the most frequently represented addictive disorder and co-occurring psychiatric condition. Multiple drug use is the most common addiction-related disorder in our sample. This was reported in four out of eight groups. These findings correspond particularly with the conclusions drawn by Cheung and Halladay [6]. They thus support the hypothesis that mental health conditions correlate with polydrug use, which poses a great risk for users. Intoxication by multiple substances may cause states that are more serious than those induced by single substances and polydrug use may also complicate the treatment of substance use disorders.

The highest silhouette value was recorded for the group encompassing multiple drug use disorders and comorbid mental health conditions from among hyperkinetic disorders. The same substance use disorder also appeared in groups with comorbid conduct disorders, neurotic, stress-related, and somatoform disorders, and behavioural syndromes associated with physiological disturbances and physical factors. While not corresponding with the results of the study by Wolitzky-Taylor et al. [5], these conclusions were in line with the findings reported by Cheung and Halladay [6]. Our conclusions highlight a high risk of multiple drug use for people with hyperkinetic disorders and conduct disorders. It is therefore important to support the early and thorough assessment of children who display symptoms of the above disorders on the one hand, and of children and adolescents with signs of polydrug use on the other hand. A comprehensive approach to the treatment of those individuals may improve its outcomes.

A limitation of this study may be the statistical methods that were chosen. The data were collected by clinicians (psychiatrist, two psychologists and supervision from another psychiatrist) working in an outpatient facility and they thus may have been not motivated to engage in systematic collection of data relevant to the research work. A significant limitation of this study is potential diagnostic bias. For instance, diagnoses like conduct disorder might be susceptible to hasty and imprecise assessments. Another limitation concerns the grouping of diagnoses in the analysis. While statistically sound, these groupings may obscure underlying heterogeneity within diagnostic categories. In addition, ICD-10 was used as the only diagnostic tool. This study was retrospective analysis and the patients’ diagnoses were not validated by any other diagnostic instruments and other checks and balances around diagnosis. Other limitations of the study include a relatively small sample and the fact that data from only one outpatient service were used. However consistent, the data for the present study were collected within a single facility and the diagnoses were made by one psychiatrist. No comparison with other services working with the same database record was made. Our research sample included adolescents aged 18, which may have led to diagnoses typically applied to adults. This finding highlights the need for further discussion regarding the upper age limit of adolescence in the context of mental health diagnoses. In samples with older adolescents, diagnoses used for adults may be more prevalent. Another limitation is that diagnoses were made by a single physician, albeit in collaboration with other mental health professionals. To reduce potential diagnostic bias, we recommend implementing a more rigorous tracking system for future studies.

Addiction-related disorders are largely preventable. Early diagnosis and widespread screening for risky behaviours are crucial, as such behaviours can exacerbate the prognosis of co-occurring psychiatric illnesses. Our study contributes to addressing the research gap in this target population, potentially informing the development of national and international clinical guidelines. Furthermore, we emphasize the importance of integrating paedopsychiatric care into addiction treatment programmes for children and adolescents. We encourage collaboration and dialogue between paedopsychiatrists and addiction specialists within services treating this population. Future research should explore the influence of family background, composition, and other contextual factors on addiction-related disorders.

## 5. Conclusions

Mental health comorbidities were highly prevalent (39%) in this sample of patients an outpatient addiction treatment clinic for children and adolescents. Psychiatric comorbidities were very often associated with multiple substance use disorders among adolescents who received treatment from the facility. Numerous researchers argue that this relationship involves an increased risk of poorer treatment outcomes among these patients. Rather than focusing on single substances, preventive and treatment interventions should follow a more comprehensive approach. The high prevalence of mental health conditions involving conduct disorders and disturbances of activity and attention suggests a high risk of substance use disorders. Prevention and early intervention for children with these conditions should therefore be recommended.

## Figures and Tables

**Table 1:** Types of mental health disorders and their representation in dual diagnoses.

Psychiatric Comorbidity		F20–F29	F30–F39	F40–F48	F50–F59	F60–F69	F70–F79	F80–F89	F90–F98
**Patients in total**	**Abs.**	1	6	16	14	22	1	12	93
	**Rel. (%)**	0.7	4.1	11.3	9.6	15.5	0.7	8.9	64.1
**Boys**	**Abs.**	1	1	6	4	11	1	8	73
	**Rel. (%)**	100	16.7	37.5	28.6	50	100	66.7	80.2
**Girls**	**Abs.**	0	5	10	10	11	0	4	20
	**Rel. (%)**	0	83.3	62.5	71.4	50	0	33.3	19.8

F20–F29 Schizophrenia, schizotypal, and delusional disorders; F30–F39 Mood (affective) disorders; F40–F48 Neurotic, stress-related, and somatoform disorders; F50–F59 Behavioural syndromes associated with physiological disturbances and physical factors; F60–F69 Disorders of adult personality and behaviour; F70–F79 Mental retardation; F80–F89 Disorders of psychological development; F90–F98 Behavioural and emotional disorders with onset usually occurring in childhood and adolescence.

**Table 2:** Summary of psychiatric diagnoses co-occurring with substance use disorders.

Substance Use Diagnosis/Psychiatric Diagnosis	F10	F12	F15	F19
**F20–F29 N (%)**	0 (0)	1 (0.6)	0 (0)	0 (0)
**F30–39 N (%)**	2 (1.1)	0 (0)	0 (0)	2 (1.1)
**F40–49 N (%)**	0 (0)	2 (1.1)	2 (1.1)	7 (4)
**F50–59 N (%)**	0 (0)	2 (1.1)	2 (1.1)	5 (2.9)
**F638 N (%)**	1 (0.6)	5 (2.9)	0 (0)	3 (1.7)
**F60–F69 N (%)**	4 (2.3)	5 (2.9)	1 (0.6)	5 (2.9)
**F70–79 N (%)**	0 (0)	0 (0)	0 (0)	1 (0.6)
**F80–F89 N (%)**	1 (0.6)	4 (2.3)	1 (0.6)	1 (0.6)
**F900–F909 (%)**	1 (0.6)	10 (5.7)	2 (1.1)	12 (6.8)
**F910–F929 N (%)**	2 (1.1)	13 (7.4)	3 (1.7)	13 (7.4)
**F93–F98 N (%)**	0 (0)	1 (0.6)	2 (1.1)	4 (2.3)

F20–F29 Schizophrenia‚ schizotypal, and delusional disorders; F30–F39 Mood (affective) disorders; F40–F49 Neurotic, stress-related, and somatoform disorders; F50–F59 Behavioural syndromes associated with physiological disturbances and physical factors; F60–F69 Disorders of adult personality and behaviour; F70–F79 Mental retardation; F80–F89 Disorders of psychological development; F900–F909 Hyperkinetic disorders; F910–F929 Conduct disorders; F93–F98 the remaining conditions belonging to behavioural and emotional disorders with onset usually occurring in childhood and adolescence.

**Table 3:** Summary of psychiatric diagnoses co-occurring with substance use disorders by gender.

Substance Use Diagnosis/Psychiatric Diagnosis	Gender	F10	F12	F15	F19
**F20–F29**	**Boys N (%)**	0 (0)	1 (0.6)	0 (0)	0 (0)
	**Girls N (%)**	0 (0)	0 (0)	0 (0)	0 (0)
**F30–39**	**Boys N (%)**	0 (0)	0 (0)	0 (0)	0 (0)
	**Girls N (%)**	2 (1.1)	0 (0)	0 (0)	2 (1.1)
**F40–49**	**Boys N (%)**	0 (0)	1 (0.6)	0 (0)	3 (1.7)
	**Girls N (%)**	0 (0)	1 (0.6)	2 (1.1)	4 (2.3)
**F50–59**	**Boys N (%)**	0 (0)	0 (0)	0 (0)	0 (0)
	**Girls N (%)**	0 (0)	2 (1.1)	2 (1.1)	5 (2.9)
**F638**	**Boys N (%)**	1 (0.6)	5 (2.9)	0 (0)	2 (1.1)
	**Girls N (%)**	0 (0)	0 (0)	0 (0)	0 (0)
**F60–F69**	**Boys N (%)**	1 (0.6)	2 (1.1)	0 (0)	1 (0.6)
	**Girls N (%)**	3 (1.7)	3 (1.7)	1 (0.6)	5 (2.9)
**F70–79**	**Boys N (%)**	0 (0)	0 (0)	0 (0)	1 (0.6)
	**Girls N (%)**	0 (0)	0 (0)	0 (0)	0 (0)
**F80–F89**	**Boys N (%)**	1 (0.6)	3 (1.7)	0 (0)	1 (0.6)
	**Girls N (%)**	0 (0)	1 (0.6)	1 (0.6)	0 (0)
**F900–F909**	**Boys N (%)**	1 (0.6)	10 (5.7)	2 (1.1)	12 (6.8)
	**Girls N (%)**	0 (0)	0 (0)	0 (0)	0 (0)
**F910–F929**	**Boys N (%)**	1 (0.6)	9 (5.1)	1 (0.6)	6 (3.4)
	**Girls N (%)**	1 (0.6)	4 (2.3)	2 (1.1)	7 (4)
**F93–F98**	**Boys N (%)**	0 (0)	1 (0.6)	0 (0)	1 (0.6)
	**Girls N (%)**	0 (0)	0 (0)	2 (1.1)	3 (1.7)

F20–F29 Schizophrenia‚ schizotypal and delusional disorders; F30–F39 Mood (affective) disorders; F40–F49 Neurotic, stress-related, and somatoform disorders; F50–F59 Behavioural syndromes associated with physiological disturbances and physical factors; F60–F69 Disorders of adult personality and behaviour; F70–F79 Mental retardation; F80–F89 Disorders of psychological development; F900–F909 Hyperkinetic disorders; F910–F929 Conduct disorders; F93–F98 The remaining conditions belonging to behavioural and emotional disorders with onset usually occurring in childhood and adolescence.

## Data Availability

Original papers included in the article could be found in the EBSCO, Medline, Scopus, and Web of Science databases.

## References

[1] Fridell M., Nilson M. (2004). Co-morbidity-drug use and mental disorders. Drugs Focus.

[2] Miovský M. (2018). Diagnostika a Terapie ADHD.

[3] Belošević M., Ferić M. (2022). Contribution of Socio-Demographic and Structured Leisure Activities’ Characteristics to Adolescents’ Alcohol Use. Adiktologie J..

[4] Tejeda-Romero C., Kobashi-Margáin R.A., Alvarez-Arellano L., Corona J.C., González-García N. (2018). Differences in Substance Use, Psychiatric Disorders and Social Factors between Mexican Adolescents and Young Adults: Dual Diagnosis in Mexican Adolescents. Am. J. Addict..

[5] Wolitzky-Taylor K., Bobova L., Zinbarg R.E., Mineka S., Craske M.G. (2012). Longitudinal Investigation of the Impact of Anxiety and Mood Disorders in Adolescence on Subsequent Substance Use Disorder Onset and Vice Versa. Addict. Behav..

[6] Cheung A.H., Cook S., Kozloff N., Chee J.N., Mann R.E., Boak A. (2019). Substance Use and Internalizing Symptoms among High School Students and Access to Health Care Services: Results from a Population-Based Study. Can. J. Public Health.

[7] Halladay J., Woock R., El-Khechen H., Munn C., MacKillop J., Amlung M., Ogrodnik M., Favotto L., Aryal K., Noori A. (2020). Patterns of Substance Use among Adolescents: A Systematic Review. Drug Alcohol Depend..

[8] Hawke L.D., Koyama E., Henderson J. (2018). Cannabis Use, Other Substance Use, and Co-Occurring Mental Health Concerns among Youth Presenting for Substance Use Treatment Services: Sex and Age Differences. J. Subst. Abus. Treat..

[9] Tomáš J., Lenka Š. (2023). Prevalence of Dual Diagnoses among Children and Adolescents with Mental Health Conditions. Children.

[10] Blevins C.E., Grimone K.R., Caviness C.M., Stein M.D., Abrantes A.M. (2019). Categorizing Cannabis and Alcohol Use Patterns of Emerging Adults in Psychiatric Partial Hospitalization Treatment. J. Psychiatr. Pract..

[11] Gobbi G., Atkin T., Zytynski T., Wang S., Askari S., Boruff J., Ware M., Marmorstein N., Cipriani A., Dendukuri N. (2019). Association of Cannabis Use in Adolescence and Risk of Depression, Anxiety, and Suicidality in Young Adulthood: A Systematic Review and Meta-Analysis. JAMA Psychiatry.

[12] Jacobus J., Squeglia L.M., Escobar S., McKenna B.M., Hernandez M.M., Bagot K.S., Taylor C.T., Huestis M.A. (2017). Changes in Marijuana Use Symptoms and Emotional Functioning over 28-Days of Monitored Abstinence in Adolescent Marijuana Users. Psychopharmacology.

[13] Mammen G., Rueda S., Roerecke M., Bonato S., Lev-Ran S., Rehm J. (2018). Association of Cannabis With Long-Term Clinical Symptoms in Anxiety and Mood Disorders: A Systematic Review of Prospective Studies. J. Clin. Psychiatry.

[14] Moitra E., Anderson B.J., Stein M.D. (2016). Reductions in Cannabis Use Are Associated with Mood Improvement in Female Emerging Adults. Depress. Anxiety.

[15] Ball J., Grucza R., Livingston M., ter Bogt T., Currie C., de Looze M. (2023). The Great Decline in Adolescent Risk Behaviours: Unitary Trend, Separate Trends, or Cascade?. Soc. Sci. Med..

[16] Miovský M., Šťastná L., Popov P. (2016). Outpatient Addiction Treatment Clinic for Children and Adolescents: Programme and Activity Structure Model: Model Struktury Programu a činnosti Ambulance Dětské a Dorostové Adiktologie. Addictology/Adiktologie.

[17] World Health Organization (WHO) (1993). The ICD-10 Classification of Mental and Behavioural Disorders.

[18] Couwenbergh C., van den Brink W., Zwart K., Vreugdenhil C., van Wijngaarden-Cremers P., van der Gaag R.J. (2006). Comorbid psychopathology in adolescents and young adults treated for substance use disorders: A review. Eur. Child Adolesc. Psychiatry.

[19] Hendl J. (2015). Přehled Statistických Metod Zpracování Dat: Analýza a Metaanalýza Dat.

